# Recurrent Revascularization Failure in a Patient with Complex Coronary Substrate

**DOI:** 10.3390/jcm15197710

**Published:** 2026-10-06

**Authors:** Nicoleta-Monica Popa-Fotea, Mircea Bajdechi, Radu-Gabriel Vătășescu, Cristina Madalina Pascu, Lucian Dorobanţu, Lucian Câlmâc

**Affiliations:** 1 Department of Cardiology, Emergency Clinical Hospital of Bucharest, 8, Calea Floreasca, 014461 Bucharest, Romania; fotea.nicoleta@yahoo.com (N.-M.P.-F.);; 2 Cardio-Thoracic Department, University of Medicine and Pharmacy “Carol Davila”, 8, Blvd., Eroii Sanitari, 050474 Bucharest, Romania; 3 Cardiology Department, Faculty of Medicine, University Ovidius, 124, Blvd. Mamaia, 900527 Constanta, Romania; 4 Cardiology Department, Emergency Clinical County Hospital, 145, Blvd. Tomis, 900591 Constanta, Romania; 5 Cardiology Department, Nord Hospital, 18, Blvd. Dimitrie Pompeiu, 020335 Bucharest, Romania

**Keywords:** in-stent restenosis, left main coronary artery, ischemia-guided PCI, intravascular imaging, bifurcation disease, diabetes mellitus, revascularization failure

## Abstract

**Background:** In-stent restenosis (ISR) remains a major limitation of contemporary percutaneous coronary intervention (PCI), particularly in patients with diabetes mellitus and complex coronary anatomy. In addition to anatomical and metabolic factors, inappropriate lesion selection and preparation may contribute to adverse outcomes and repeated revascularization. **Case Summary:** A 44-year-old man with insulin-treated type 2 diabetes mellitus, arterial hypertension, and dyslipidemia presented with non-ST-segment elevation myocardial infarction. His recent cardiovascular history included multiple PCI procedures with drug-eluting stent (DES) implantation in the left anterior descending and circumflex arteries. Early recurrent ISR developed and progressed to involve the left main coronary artery (LMCA) bifurcation, with rapid evolution toward multilayer restenosis despite repeat drug-coated balloon (DCB) angioplasty and additional stent implantation. Given the complex coronary anatomy (residual SYNTAX score 32) and repeated PCI failure, the Heart Team recommended coronary artery bypass grafting. However, early graft degeneration and progression of native coronary disease led to recurrent angina. Subsequent intravascular ultrasound (IVUS)-guided PCI using a hybrid strategy (DCB and DES) resulted in favorable short-term outcomes, with no significant restenosis at 6- and 12-month follow-up. **Conclusions**: This case illustrates how recurrent restenosis may develop despite ischemia-supported PCI when complex bifurcation anatomy and mechanical factors such as stent underexpansion remain insufficiently characterized. In complex coronary disease, systematic use of intracoronary imaging and mechanism-based revascularization are essential to prevent repeated target lesion failure.

## 1. Introduction

In-stent restenosis (ISR) remains a clinically relevant limitation of contemporary percutaneous coronary intervention (PCI), particularly in patients with diabetes mellitus and complex coronary anatomy [1,2]. Although new-generation drug-eluting stents (DES) have reduced restenosis rates, ISR continues to occur in challenging settings such as bifurcation lesions and left main coronary artery (LMCA) disease [1].

Current guidelines emphasize that revascularization decisions should not rely solely on angiographic appearance, but should be supported by objective evidence of myocardial ischemia [3]. Physiologic assessment using fractional flow reserve (FFR) or instantaneous wave-free ratio (iFR), together with intracoronary imaging—intravascular ultrasound (IVUS) or optical coherence tomography (OCT), plays a central role in both lesion selection and procedural optimization [3].

However, angiography-guided PCI without functional confirmation remains common in clinical practice [3]. This approach may lead to unnecessary stent implantation, particularly in anatomically complex regions such as the LMCA bifurcation, potentially initiating a cascade of adverse events including multilayer stenting, recurrent ISR, and repeated revascularization [3,4].

We present a case illustrating how complex bifurcation anatomy, recurrent mechanical restenosis, and lack of intracoronary imaging during earlier interventions may contribute to progressive revascularization failure.

## 2. Case Presentation

This case report was prepared in accordance with the CARE (CAse REport) guidelines. The completed CARE checklist is provided in the Supplementary Materials (Table S1). A 44-year-old man with multiple cardiovascular risk factors, including arterial hypertension, insulin-treated type 2 diabetes mellitus, and dyslipidemia, presented in July 2025 with acute retrosternal chest pain at rest. The pain was constrictive in character and lasted more than 30 min. He denied dyspnea, palpitations or syncope.

On admission, the patient was hemodynamically stable. Blood pressure and heart rate were within normal limits. Cardiac auscultation revealed normal heart sounds without murmurs. There were no signs of heart failure, including jugular venous distention, peripheral edema, or pulmonary crackles.

The 12-lead electrocardiogram demonstrated sinus bradycardia (50 beats per minute) with left axis deviation. Mild ST-segment depression and T-wave inversion were observed in leads I, aVL, V6, and II, consistent with lateral subendocardial ischemia.

Laboratory testing showed elevated cardiac biomarkers, with high-sensitivity troponin I measuring 630 ng/L (reference < 40 ng/L), confirming acute myocardial necrosis. NT-pro-BNP was elevated at 1200 pg/mL (reference < 150 ng/L), indicating increased myocardial wall stress. Renal function was preserved (serum creatinine 99 µmol/L, reference 53–97 ng/L).

**Figure 1:**
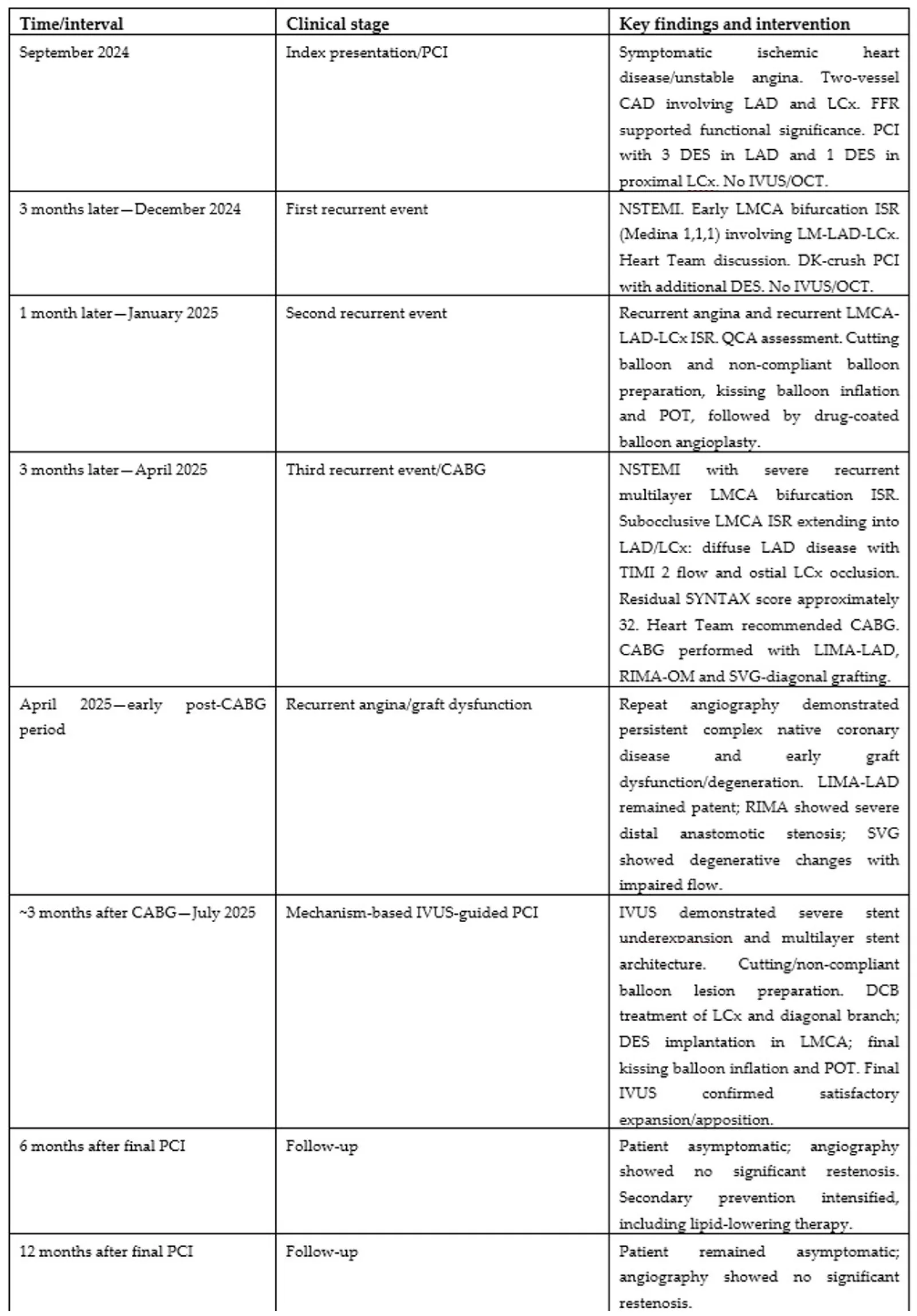
CARE Clinical Flow Diagram. CABG, coronary artery bypass grafting; CAD, coronary artery disease; DCB, drug-coated balloon; DES, drug-eluting stent; FFR, fractional flow reserve; IVUS, intravascular ultrasound; LAD, left anterior descending artery; LCx, left circumflex artery; LIMA, left internal mammary artery; LMCA, left main coronary artery; OCT, optical coherence tomography; OM, obtuse marginal branch; POT, proximal optimization technique; QCA, quantitative coronary angiography; RIMA, right internal mammary artery; SVG, saphenous vein graft.

**Figure 2:**
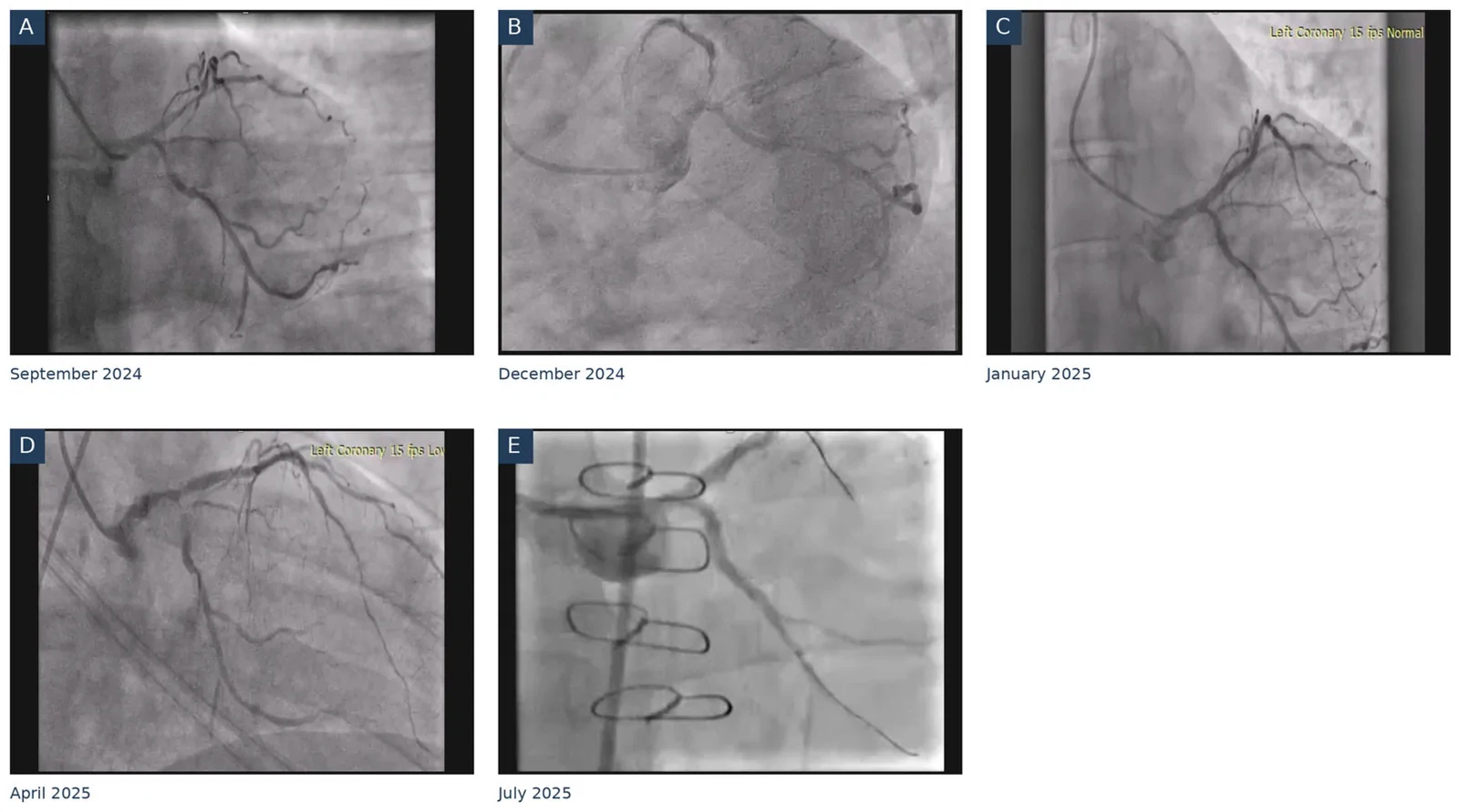
Serial coronary angiographic findings. Representative frames from (**A**) September 2024, (**B**) December 2024, (**C**) January 2025, (**D**) April 2025, and (**E**) July 2025 illustrate the course of coronary disease and successive revascularization procedures. The corresponding dynamic angiographic sequences are provided in the Supplementary Videos.

His cardiovascular history (Figure 1 and Figure 2) began in September 2024, when the patient was hospitalized for acute coronary syndrome–unstable angina; the coronary angiography performed at that time demonstrated significant two-vessel coronary artery disease, with severe stenotic lesions involving the proximal and mid left anterior descending artery (LAD) and the proximal left circumflex (LCx) (Video S1). The complete angiographic sequence is provided to allow assessment of lesion morphology, vessel filling, and the extent of coronary involvement. The baseline anatomical SYNTAX score was 16, consistent with low anatomical complexity. The case was discussed by the multidisciplinary Heart Team, and both surgical and percutaneous revascularization strategies were considered, particularly in view of the patient’s young age and diabetes mellitus. However, the coronary anatomy was considered technically suitable for PCI, with no left main disease, chronic total occlusion, or complex bifurcation involvement. After being informed about the relative benefits and limitations of PCI and CABG, the patient expressed a clear preference for percutaneous revascularization. Accordingly, the Heart Team supported PCI as the initial revascularization strategy. Physiological lesion assessment using FFR was performed before the index PCI in September 2024. Measurements were obtained during adenosine-induced hyperemia, with the pressure wire positioned in the distal portion of each interrogated vessel. Distal FFR values were 0.68 in the LAD and 0.72 in the LCx, confirming the functional significance of disease in both vessels. Pressure-wire pullback demonstrated a predominantly diffuse pressure loss through the mid and proximal LAD, consistent with the long angiographic disease burden, whereas the LCx showed a predominantly focal pressure drop corresponding to the proximal lesion. A formal pullback pressure gradient index was not calculated. Pressure-wire drift was assessed after pullback at the guiding catheter tip and was absent.

PCI was undertaken with implantation of three DESs in the LAD, from the distal to the proximal segment (XIENCE PRO 2.75 × 28 mm, XIENCE PRO 3.0 × 23 mm, and XIENCE PRO 3.5 × 28 mm, respectively), and one XIENCE PRO drug-eluting stent (3.0 × 18 mm) in the proximal LCx. Intracoronary imaging (IVUS/OCT) was not used during the index intervention. In December 2024, the patient presented directly to the hospital following the onset of acute chest pain and was diagnosed with non ST-elevation myocardial infarction (NSTEMI) (peak hs-cTnI 630 ng/L (reference < 40 ng/L). Transthoracic echocardiography demonstrated mildly reduced left ventricular (LV) systolic function, with a LV ejection fraction (LVEF) of 45%. The LV was non-dilated, with mild regional hypokinesis involving the anterior, anterolateral and anteroseptal walls and no significant valvular disease was identified. The distribution of the regional wall-motion abnormalities was consistent with involvement of the LAD and LCX territory. Coronary angiography performed during the acute presentation demonstrated early ISR involving the left main coronary artery (LMCA) bifurcation, with a true Medina 1,1,1 pattern involving the LAD and LCx and severely impaired antegrade flow in the LAD (TIMI 1) (Video S3). Although the patient remained hemodynamically stable, the acute coronary syndrome presentation, complex LMCA bifurcation restenosis, and markedly impaired LAD flow prompted urgent revascularization during the same clinical episode. Given the significant involvement of both branches of the true LMCA bifurcation, a planned two-stent strategy was selected, and PCI was performed using the DK-crush technique. Lesion preparation of the LAD included predilatation with a semi-compliant balloon (SCB) 3.5 × 20 mm, followed by a 3.5 × 15 mm Wolverine cutting balloon and a non-compliant balloon (NCB) 4.0 × 15 mm. The LCx was predilated with a SCB 3.0 × 20 mm, followed by a NCB 3.5 × 15 mm. A XIENCE PRO S 4.0 × 18 mm DES was implanted from the LAD into the LMCA, and a XIENCE PRO S 3.5 × 18 mm DES was implanted in the LCx. Final kissing-balloon inflation was performed using a NCB 4.0 × 12 mm in the LAD and a NCB 3.5 × 12 mm in the LCx, followed by final proximal optimization technique (POT) of the LMCA using a NCB 5.5 × 8 mm. Intravascular imaging was not performed during this procedure, limiting mechanistic assessment of ISR at that stage. In January 2025, the patient was readmitted with recurrent chest pain and unstable angina. hs-cTnI remained within the normal reference range, with no biochemical evidence of acute myocardial injury. Recurrent ISR involving the LMCA–LAD–LCx bifurcation was diagnosed angiographically and quantified using quantitative coronary angiography. Given the presence of multiple previously implanted stent layers and the desire to avoid further metallic burden, a drug-coated balloon (DCB) strategy was selected. Lesion preparation was performed using a cutting balloon Wolverine 3.5 × 10 mm in the LAD and 3.0 × 10 mm in the LCx, followed by NCB dilatation with a 4.0 × 12 mm balloon in the LAD and a 3.5 × 12 mm in the LCx. Subsequently, DCB angioplasty was performed using a SeQuent Please NEO DCB 4.0 × 20 mm in the LAD and a 3.5 × 20 mm SeQuent Please NEO DCB in the LCx. Procedural optimization included kissing-balloon inflation using a 3.5 × 15 mm balloon in the LAD and a 3.0 × 15 mm balloon in the LCx, followed by POT with a NCB 5.5 × 8 mm. The mechanism of recurrent ISR and the degree of stent expansion could not be directly assessed at this stage as intracoronary imaging (IVUS/OCT) was not performed.

In April 2025, the patient presented with recurrent angina and elevated hs-cTnI to 450 ng/L (upper reference limit < 40 ng/L), consistent with acute myocardial injury in the setting of NSTEMI. Coronary angiography (Video S3) demonstrated subocclusive LMCA in-stent restenosis extending into the proximal LAD and LCx. The complete sequence additionally demonstrates diffuse LAD disease with near-ostial subocclusion and impaired distal flow (TIMI 2), ostial LCx occlusion due to ISR, and the dynamic filling of the remaining coronary circulation. The right coronary artery was diffusely diseased without significant stenosis.

**Figure 3:**
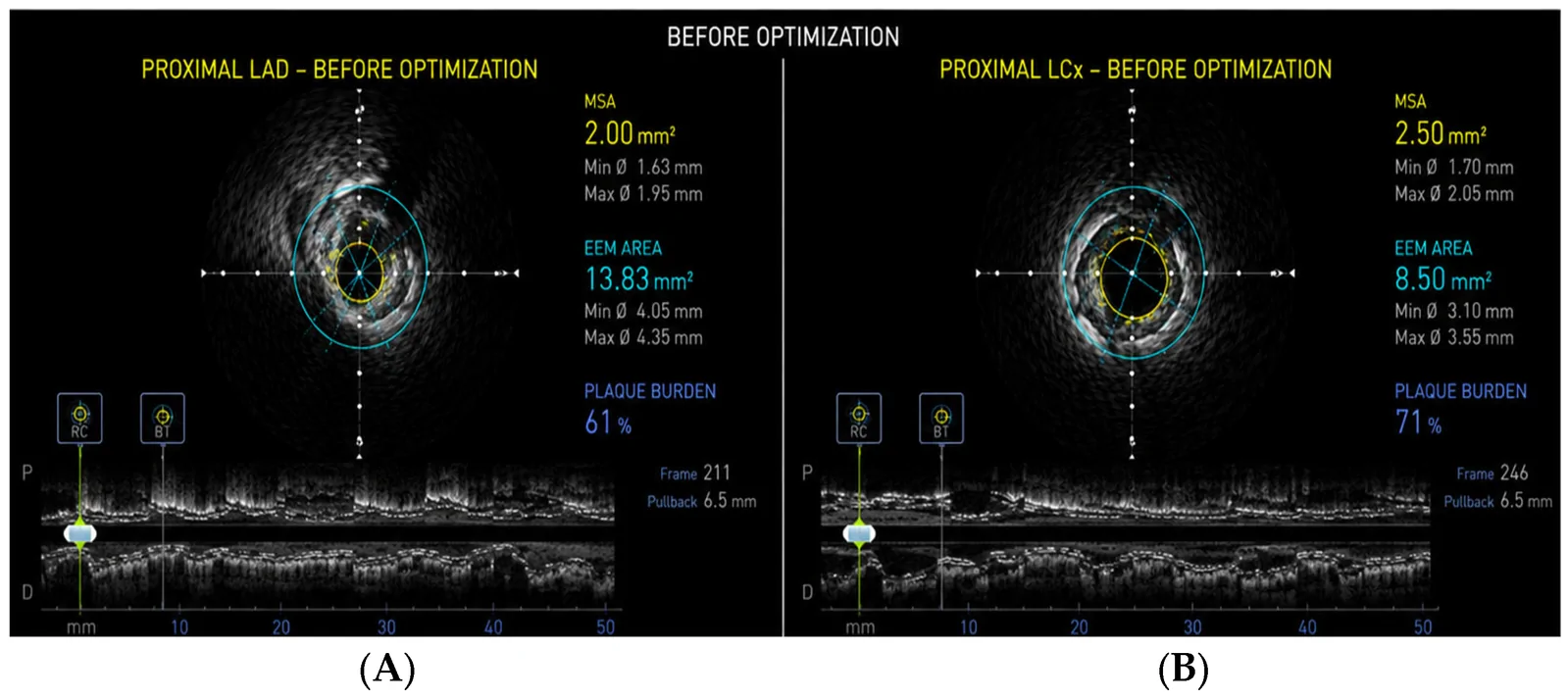
IVUS imaging of the LMCA–LAD (**A**) and LMCA-LCX (**B**) stent showing significant stent underexpansion before optimization. MSA minimum stent area; EEM external elastic membrane; plaque burden = (EEM area − lumen area)/EEM area × 100(%).

Based on the angiographic findings at the time of presentation, the residual SYNTAX score was independently assessed by two experienced interventional cardiologists, who reached a concordant estimate of 32, supporting substantial residual anatomical disease and revascularization complexity. Given the combination of diabetes mellitus, complex LMCA bifurcation disease, and repeated revascularization failure, the case was discussed by a multidisciplinary Heart Team and coronary artery bypass grafting (CABG) was decided. Before CABG, three-dimensional echocardiographic assessment demonstrated moderately reduced LVEF, with an LVEF of 40%. The LV remained non-dilated, with mild regional hypokinesis involving the anterior, anteroseptal, and anterolateral walls. No significant valvular disease was identified. CABG was subsequently performed using a left internal mammary artery (LIMA) graft to the LAD, together with a right internal mammary artery (RIMA) to the obtuse marginal and an inverted saphenous venous (SVG) graft to the first diagonal. Several months later, in July 2025, recurrent angina prompted repeat coronary angiography. At the subsequent presentation with recurrent symptoms, hs-cTnI remained within the normal reference range, with no biochemical evidence of acute myocardial injury. Following the recurrence of symptoms, transthoracic echocardiography demonstrated a LVEF of 40%. The LV remained non-dilated, with mild regional hypokinesis involving the anterior, anteroseptal, and anterolateral walls. No further deterioration in global left ventricular systolic function was observed compared with the pre-CABG assessment. The complete angiographic sequence was particularly useful for assessing both native coronary circulation and graft function (Video S3). Angiography demonstrated patent bypass grafts, but important limitations of graft-dependent myocardial perfusion. The LIMA–LAD graft was patent, although the distal native LAD was diffusely affected by atherosclerotic disease. The graft supplying the LCx/OM territory was patent but showed severe stenosis at the distal anastomosis, while the native LCx was chronically occluded in its proximal segment, and the SVG supplying the diagonal territory exhibited degenerative changes with impaired flow. These findings represented heterogeneous mechanisms of impaired graft-dependent perfusion rather than uniform graft occlusion. Quantitative target-vessel diameters and formal distal-runoff measurements were not available, and no definitive angiographic evidence established competitive flow as the mechanism of graft dysfunction. Accordingly, poor runoff, competitive flow, and diabetes mellitus were not considered proven causes of the observed graft abnormalities. Following successful antegrade recanalization of the chronically occluded proximal LCx, IVUS demonstrated a complex multilayer stent architecture involving the LMCA bifurcation, with severe underexpansion of the previously implanted stents. The minimum stent area (MSA) was approximately 2.0 mm^2^ in the proximal LAD and 2.5 mm^2^ in the ostial/proximal LCx component of the LM–LCx pathway (Figure 3). The corresponding reference lumen areas were approximately 13.8 mm^2^ in the LAD and 8.5 mm^2^ in the LCx, while the distal LMCA reference lumen area was approximately 22 mm^2^. Underexpansion extended across the previously treated bifurcation segments and was most pronounced at the proximal LAD and ostial/proximal LCx. The marked discrepancy between the MSA and the corresponding reference lumen dimensions identified severe mechanical stent underexpansion within the multilayer bifurcation construct as a major substrate for recurrent ISR and provided the rationale for intensive lesion preparation and IVUS-guided optimization.

**Figure 4:**
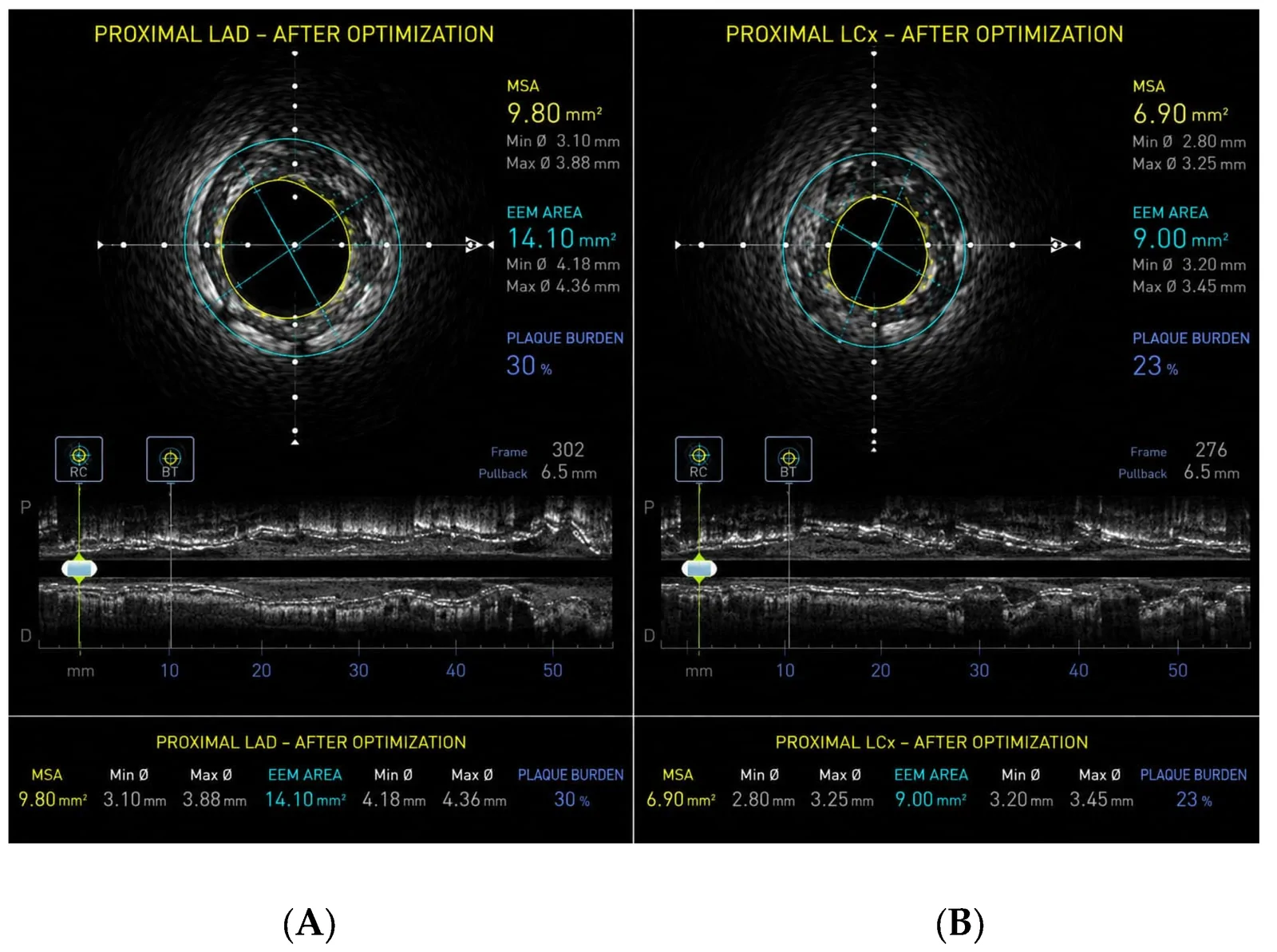
IVUS imaging of the LMCA–LAD (**A**) and LMCA-LCX (**B**) stent after optimization. MSA minimum stent area; EEM external elastic membrane; plaque burden = (EEM area − lumen area)/EEM area × 100(%).

The final revascularization strategy was individualized according to the anatomical and IVUS-defined mechanism in each segment, with the aim of correcting mechanical underexpansion while minimizing further metallic burden. In the LAD, severe underexpansion of the previously implanted stents was treated with a cutting balloon Wolverine 4.0 × 15 mm followed by a NCB 4.0 × 18 mm, without implantation of an additional stent. Following successful antegrade recanalization of the chronically occluded proximal LCx and intensive lesion preparation with a cutting balloon Wolverije 3.5 × 15 mm and a NCB 3.5 × 15 mm, DCB 3.5 × 20 mm SeQuent Please NEO was used to deliver antiproliferative therapy while avoiding unnecessary additional metallic layering. The severely diseased diagonal branch was similarly treated with a DCB 2.75 × 25 mm SeQuent Please NEO, particularly given the degenerative SVG with impaired flow to this territory. Additional DES implantation was deliberately restricted to the LMCA–LCx segment, where a 4.0 × 18 mm XIENCE PRO S DES was considered necessary to provide adequate radial support and stabilize the newly recanalized LMCA-to-LCx pathway after extensive lesion preparation. The procedure was completed with final kissing-balloon inflation using a NCB 4.5 × 12 mm balloon in the LAD and a SCB 4.0 × 12 mm balloon in the LCx, followed by POT of the LMCA with NCB 6.0 × 8 mm. Final IVUS assessment confirmed satisfactory stent expansion and apposition (Figure 4).

Despite treatment with atorvastatin 80 mg combined with ezetimibe 10 mg daily, LDL cholesterol remained above target at 2.49 mmol/L (<1.4 mmol/L recommended for very high-risk patients). Given persistent LDL-cholesterol levels above guideline-recommended targets despite maximally tolerated statin and ezetimibe therapy, treatment was further intensified with a PCSK9 inhibitor, LDL-C decreasing to 30 mg/dL. According to the patient’s report and available clinical records, dual antiplatelet therapy with acetylsalicylic acid and ticagrelor was continued throughout follow-up; however, adherence was not objectively verified. Blood pressure was adequately controlled during follow-up. No major or clinically relevant bleeding events were recorded during re-evaluations. Diabetes treatment consisted of metformin, an SGLT2 inhibitor, and insulin therapy, with HbA1c values around 6.7%, indicating acceptable glycemic control. Left ventricular systolic function remained stable, with a LVEF of approximately 40%. No formal non-invasive ischemia testing was performed during follow-up. At 6 and 12-month, the patient remained asymptomatic, and invasive coronary angiography performed as part of an individualized surveillance strategy rather than as routine follow-up after uncomplicated PCI showed no significant lesions.

## 3. Discussion

This case highlights the complex interplay between procedural strategy, anatomical complexity, and metabolic risk factors in recurrent revascularization failure. A key feature of this case is that recurrent restenosis developed despite physiologically supported PCI performed in the setting of acute coronary syndrome. The subsequent clinical course highlights that, beyond lesion selection, procedural optimization and identification of mechanical determinants of failure are critical in complex bifurcation disease.

Contemporary management of recurrent DES-ISR is increasingly based on identification and correction of the underlying mechanism. Current ESC recommendations support IVUS- or OCT-guided PCI for anatomically complex lesions, including LMCA, true bifurcation, and long lesions (Class I, Level of Evidence A) [5]. In parallel, the EAPCI expert consensus on myocardial revascularization failure and the SCAI Expert Consensus Statement on ISR emphasize intracoronary imaging for determining the mechanism of stent failure and guiding subsequent therapy [6]. IVUS and OCT can identify mechanical causes such as stent underexpansion, malapposition, fracture or deformation, and geographic miss, and distinguish these from biological mechanisms including neointimal hyperplasia and neoatherosclerosis. This distinction has direct therapeutic implications: significant underexpansion requires correction of the mechanical constraint and adequate lesion preparation before additional antiproliferative therapy is considered. The EAPCI consensus specifically advocates aggressive high-pressure non-compliant balloon dilatation for underexpanded stents, with escalation to dedicated plaque-modification techniques when underexpansion is resistant, followed by DES or DCB treatment after satisfactory lesion preparation [6].

The number of pre-existing metallic layers is another important consideration in recurrent ISR. Although repeat DES implantation remains an effective treatment for ISR, further stent layers may perpetuate underexpansion, increase procedural complexity, compromise side-branch access, and provide an additional substrate for recurrent restenosis. The EAPCI consensus therefore highlights DCB as an attractive option when multiple metallic layers are already present, because it delivers antiproliferative therapy without introducing an additional scaffold [6]. In patients with recurrent episodes of diffuse ISR, CABG or conservative management should also be considered rather than repeated PCI following Heart Team discussion [6]. These considerations were particularly relevant in the present case, in which IVUS demonstrated severe underexpansion within a complex multilayer LMCA bifurcation construct. The final intervention therefore prioritized aggressive lesion preparation and IVUS-guided optimization while minimizing additional metallic burden: DCB angioplasty was used where feasible, whereas additional DES implantation was restricted to the LMCA–LCx segment where further radial support was considered necessary.

Importantly, the initial decision to perform revascularization should be interpreted in the context of acute coronary syndrome, physiologically significant LAD and LCx lesions confirmed by FFR, insulin-treated diabetes mellitus, and multivessel coronary artery disease. Contemporary revascularization guidelines recognize both anatomical complexity and clinical presentation as important determinants of revascularization strategy selection [7,8]. In such patients, both PCI and CABG may represent reasonable treatment options depending on coronary anatomy, clinical stability, and Heart Team assessment [7,8]. The physiological pattern of disease is also relevant when interpreting the initial revascularization strategy. As recently emphasized by Sparasci et al., longitudinal pressure-wire pullback provides information beyond a single distal FFR measurement by characterizing the distribution of epicardial pressure loss and distinguishing predominantly focal from diffuse coronary disease [9]. The PPG further provides a quantitative description of this continuum, with lower values generally reflecting more diffuse disease and higher values a more focal pattern [10]. In the present case, although both vessels were physiologically significant, the LAD exhibited a predominantly diffuse pressure-loss pattern, whereas the LCx showed a predominantly focal gradient. This distinction is clinically relevant because diffuse physiological disease may limit the gain achievable with extensive stenting and should be considered when anticipating the physiological benefit of PCI.

Discordance between anatomical and functional assessment is common and is particularly relevant in left main disease. In an analysis of 4086 stenoses, classification based on at least 50% angiographic diameter stenosis disagreed with FFR of 0.80 or less in 35% of lesions, and classical angiographic thresholds performed particularly poorly in LMCA [11]. The PHYNAL registry further reported iFR/FFR discordance in 25% of patients with isolated left main disease and 36.2% in the presence of downstream disease; discordance between physiology and an IVUS minimal lumen area threshold of 6 mm^2^ occurred in 29.4–37.0% [12]. These findings should not be interpreted as evidence that one modality is intrinsically incorrect, because physiology assesses lesion-specific hemodynamic significance, whereas IVUS or OCT identifies plaque morphology, vessel dimensions, and mechanical causes of failure. In the present case, FFR-supported lesion significance at the index procedure did not exclude inadequate lesion preparation, stent underexpansion, or suboptimal bifurcation optimization—mechanisms that could only be characterized by intracoronary imaging.

ISR remains a clinically meaningful limitation of PCI despite newer-generation DES technologies, particularly in patients with diabetes mellitus and in complex anatomies such as bifurcations and left main disease [1,13]. Contemporary reviews emphasize that, although average restenosis rates have fallen, the absolute burden of ISR remains high because of the large global volume of PCI, and that an individualized, mechanism-based approach supported by intracoronary imaging is central to management [2]. Diabetes further amplifies failure risk through multiple pathways, including enhanced neointimal proliferation and altered vascular healing responses after stent implantation, translating into higher ISR risk compared with non-diabetic patients [14]. This case illustrates early, recurrent multilayer ISR involving the LMCA bifurcation in a relatively young insulin-treated diabetic patient, followed by graft degeneration after CABG.

Early ISR (<6 months) is usually related to procedural or mechanical factors such as stent underexpansion, incomplete lesion preparation, or geometric distortion rather than neoatherosclerosis [1,13]. Intravascular imaging studies consistently identify stent underexpansion as the leading mechanism of DES failure, particularly in calcified or left main lesions [15,16]. The initial revascularizations in this case were angiography-guided and not supported by intravascular imaging. Current ESC/EACTS myocardial revascularization guidelines emphasize the role of IVUS for optimization of LMCA PCI [3]. Randomized evidence supports improved clinical outcomes with IVUS-guided PCI compared with angiography guidance. In the ULTIMATE trial, target vessel failure at 3 years occurred in 6.6% of patients undergoing IVUS-guided PCI compared with 10.7% with angiography guidance (HR 0.60, 95% CI 0.42–0.87; *p* = 0.01), corresponding to an absolute risk reduction of 4.1% and an approximate number needed to treat (NNT) of 24 [16]. In the IVUS-XPL trial, 5-year major adverse cardiac events occurred in 5.6% versus 10.7% of patients, respectively (HR 0.50, 95% CI 0.34–0.75; *p* = 0.001), corresponding to an absolute risk reduction of 5.1% and an approximate NNT of 20 [17]. The benefit in IVUS-XPL was driven predominantly by a reduction in target lesion revascularization (4.8% vs. 8.4%; HR 0.54, 95% CI 0.33–0.89; *p* = 0.007) [17]. The angiographic appearance in our case was consistent with diffuse proliferative ISR (Mehran class II–III), a morphology associated with higher recurrence rates [13].

Although physiologic significance was documented, angiographic assessment alone may still have limited the ability to characterize lesion morphology, bifurcation geometry, plaque distribution, and vessel dimensions. In complex bifurcation lesions, intracoronary imaging may complement physiologic assessment by refining procedural planning and optimizing stent deployment [3,18,19]. In intermediate or angiographically ambiguous LMCA lesions, both intracoronary imaging and physiologic assessment can help determine whether revascularization is truly necessary and can refine lesion selection before stent implantation [3,18,20]. Because IVUS or OCT was not used to guide revascularization after the first ISR, the precise morphology, dimensions, and mechanical characteristics of individual lesion segments cannot be retrospectively established. Consequently, whether all treated segments represented equally relevant components of the ischemic substrate remains uncertain [2,6,18].

Although IVUS and OCT are both valuable intracoronary imaging modalities, their respective strengths should be distinguished. IVUS provides greater tissue penetration and is particularly suited to the assessment of vessel dimensions, plaque burden, deeper calcium, remodeling, stent expansion, and complex multilayer stent architecture [18]. OCT, in contrast, provides substantially higher spatial resolution and enables detailed characterization of superficial plaque and stent-related morphology, including fibrous-cap integrity, plaque rupture or erosion, thrombus, neointimal tissue, neoatherosclerosis, strut coverage, and subtle malapposition, although at the expense of lower tissue penetration and the need for transient blood clearance [18,21]. OCT has also assumed an important role in elucidating the mechanisms underlying both acute and non-acute myocardial ischemic syndromes [22]. In the present case, IVUS was particularly appropriate because the principal procedural question concerned the mechanical assessment of severe stent underexpansion, vessel dimensions, and multilayer architecture within a complex LMCA bifurcation construct. Thus, IVUS and OCT should be considered complementary rather than interchangeable techniques, with modality selection guided by the specific anatomical and mechanistic question [18,21]. Such overtreatment may increase stent length and metal burden, thereby predisposing to restenosis, particularly in small-caliber vessels and bifurcation anatomy [2,6,13]. Contemporary reviews of LMCA assessment stress that overestimation of lesion severity can lead to major but unnecessary intervention, whereas underestimation risks inappropriate deferral, highlighting the value of physiology- and imaging-guided decision-making in this territory [23].

Multilayer stenting further increases metal burden and restenosis risk, especially in bifurcation disease [1,7]. Repeated stent implantation at bifurcation sites may lead to excessive metallic layers at the carina and side-branch ostium, promoting neointimal proliferation and flow disturbance [18]. The estimated residual SYNTAX score (~32) indicated intermediate-to-high anatomical complexity. In addition, the lesion fulfilled several criteria for complex coronary bifurcation disease described in consensus documents from the European Bifurcation Club [18]. Current European and American guidelines generally support CABG as the preferred revascularization strategy in patients with diabetes mellitus, multivessel coronary artery disease, and increasing anatomical complexity, particularly when recurrent PCI failure occurs [7,8].

The extent of native-vessel revascularization after CABG deserves particular consideration because restoration of native coronary flow may theoretically increase competitive flow with patent bypass grafts and potentially influence long-term graft function. In the present case, however, native-vessel PCI was not pursued as a strategy of routine complete revascularization. Each treated territory had a specific anatomical or mechanical indication: the LM–LCx pathway showed severe IVUS-confirmed stent underexpansion after recanalization of the proximally occluded LCx (MSA 2.5 mm^2^), the LAD showed severe stent underexpansion on IVUS (MSA approximately 2.0 mm^2^), and the diagonal branch had severe proximal stenosis in the setting of a degenerative SVG with impaired flow. Moreover, graft-dependent perfusion was itself potentially limited by diffuse distal LAD disease and severe stenosis at the LCx/OM graft anastomosis. The potential for competitive flow was therefore weighed against the demonstrated limitations of both the native and surgical conduits. Importantly, additional metallic burden was minimized: LAD underexpansion was treated by balloon-based optimization without further stenting, the diagonal branch was treated with a DCB, and additional DES implantation was restricted to the LM–LCx pathway.

The present case therefore reflects not only restenosis after PCI, but also the cumulative adverse impact of repeated stent layering in a LMCA bifurcation that was anatomically complex from the outset.

Another key mechanistic consideration is incomplete lesion preparation before the initial stent implantations. In heavily diseased bifurcation and left main lesions, especially when calcium, fibrotic plaque, or vessel size mismatch are present, inadequate plaque modification may result in incomplete stent expansion [13]. Angiography frequently underestimates calcium burden and does not reliably identify eccentric plaque distribution, stent asymmetry, or insufficient minimal stent area [13]. Several interacting mechanisms may have contributed to the very early recurrence observed in this patient, including complex bifurcation anatomy, multilayer stenting, metabolic risk related to diabetes, and potentially suboptimal lesion preparation and stent expansion. The subsequent IVUS demonstration of underexpanded DES in the LMCA, LAD, and LCx confirms the presence of a mechanical abnormality; however, its relative contribution to recurrent ISR cannot be separated from these concomitant factors. The subsequent IVUS finding of underexpanded DES in the LMCA, LAD, and LCx is consistent with this proposed mechanism, although a causal relationship cannot be established retrospectively. Because intracoronary imaging was not performed during the earlier interventions, the mechanisms underlying the initial and subsequent episodes of stent failure cannot be established retrospectively. Lesion overestimation, inadequate modification of underlying calcific disease, geographic miss, and initial stent underexpansion therefore remain plausible hypotheses rather than demonstrated causes. IVUS performed during the final intervention in July 2025 directly documented severe stent underexpansion within the complex multilayer LMCA bifurcation construct, establishing an important mechanical substrate at that stage; however, these findings cannot determine whether underexpansion was already present immediately after the earlier PCI procedures. This interpretation is highly consistent with current stent failure frameworks, in which failure to achieve adequate lesion preparation and final stent expansion is a dominant substrate for early DES restenosis [2,6,13].

Graft degeneration may have been influenced by poor distal runoff, small-caliber target vessels, and competitive native coronary flow, although the relative contribution of these factors cannot be determined from this single case [2,23]. Graft failure after CABG varies substantially according to conduit type. In the PREVENT IV angiographic cohort, saphenous vein graft failure, defined as ≥75% stenosis or occlusion, was documented in 1096 of 4343 saphenous vein grafts (25.2%) assessed by protocol-mandated angiography at 12–18 months after CABG or by earlier clinically indicated angiography [24]. Arterial grafts showed lower failure rates in a separate PREVENT IV analysis: among 1539 patients with an internal mammary artery (IMA) graft to the LAD and angiographic follow-up at 12–18 months, IMA failure, similarly defined as ≥75% stenosis or occlusion, occurred in 132 patients (8.6%); 99.4% of the evaluated IMA grafts were left IMA grafts [25]. Importantly, IMA failure was associated with less severe native LAD stenosis (<75%; OR 1.76, 95% CI 1.19–2.59) and with an additional bypass graft to the diagonal branch (OR 1.92, 95% CI 1.33–2.76), findings compatible with a role for competitive flow. Longer-term observational angiographic data also demonstrate high patency of right internal thoracic artery grafts, although these symptom-driven cohorts are not directly comparable with protocol-mandated PREVENT IV angiography [26]. These data underscore that graft failure rates and potential mechanisms differ by conduit type and target-vessel characteristics. In the present case, the poor distal native-vessel bed and persistent diffuse disease likely limited the long-term benefit of surgical revascularization despite technically appropriate graft selection. Diabetes may further promote exaggerated neointimal proliferation and inflammatory vascular responses, increasing the risk of both ISR and graft failure [7,27]. Thus, we hypothesize that failure of both PCI and CABG may reflect the interaction of an exceptionally unfavorable anatomical and metabolic substrate rather than failure attributable to a single revascularization modality.

Prolonged DAPT was justified by the patient’s high ischemic risk and low bleeding risk profile, in accordance with ESC recommendations for NSTEMI [28]. Aggressive lipid-lowering therapy is also essential in this setting. ESC dyslipidemia guidelines recommend an LDL-cholesterol target <1.4 mmol/L with ≥50% reduction from baseline in very-high-risk patients, and the addition of PCSK9 inhibitors when targets are not achieved despite maximally tolerated statin and ezetimibe therapy [29]. In this case, intensified secondary prevention was particularly important because recurrent events occurred despite apparently appropriate antiplatelet therapy and standard high-intensity lipid-lowering treatment, indicating an extreme residual ischemic risk phenotype.

The recurrent ISR observed in this patient occurred in the presence of several concomitant and potentially interacting risk factors, including diabetes mellitus, complex LMCA bifurcation anatomy, repeated multilayer stenting, and the absence of intracoronary imaging during the earlier procedures. Potentially incomplete lesion preparation and suboptimal stent expansion may have represented additional contributors. Given the single-case design and the substantial interdependence among these factors, their individual effects cannot be disentangled or causally ranked [13,27,30,31].

In addition, repeated stent implantation at the LMCA bifurcation increased local metal burden and altered flow dynamics, promoting neointimal hyperplasia and recurrent restenosis in complex bifurcation lesions [13,32]. Finally, the absence of intravascular imaging during the initial procedures may have limited the recognition and correction of suboptimal stent expansion, a recognized mechanical mechanism of early DES failure [18,20]. To these factors one may reasonably add three interacting contributors: possible overestimation of the initial angiographic disease severity, incomplete lesion preparation before stent delivery, and incomplete post-dilatation/final optimization [2,3]. Together, these factors may have created the substrate for very early mechanical failure, especially in a diabetic patient with diffuse disease and unfavorable bifurcation geometry [13,32].

This case also highlights an important practical lesson: in complex LMCA bifurcation disease, the central question is not only how to treat the lesion, but whether the lesion segments selected for treatment truly require stenting and whether the vessel has been adequately prepared to receive a stent [3,19,20]. When these steps are insufficiently addressed, each subsequent reintervention may amplify future failure by increasing metallic burden and procedural complexity [8,13]. Conversely, the favorable short-term evolution observed after IVUS-guided, mechanism-based reintervention illustrates the potential value of meticulous reassessment of the failure mechanism, although no causal inference can be drawn from this single case [2,6,21].

Overall, this case highlights the complex interplay between anatomical complexity, metabolic risk factors, and procedural strategy in left main bifurcation disease. It also underscores the importance of intravascular imaging, careful avoidance of excessive metal layering, and multidisciplinary Heart Team decision-making when managing recurrent revascularization failure.

## 4. Limitations

This case report has several limitations. Therefore, the contribution of lesion overestimation, underexpansion, calcium-related mechanical resistance, geographic miss, or stent edge disease can only be inferred retrospectively. Second, histopathological confirmation of the restenotic substrate was not available, and differentiation between pure neointimal hyperplasia and early neoatherosclerotic change was based on timing and imaging context rather than tissue characterization. Third, this is a single-patient observation, so the therapeutic sequence used here cannot be generalized to all cases of recurrent LMCA bifurcation ISR. Fourth, follow-up duration remains limited, and longer clinical and imaging surveillance is needed to determine the durability of the final IVUS-guided PCI strategy. An additional limitation is the inseparable confounding inherent to this single-patient observation. Multiple anatomical, metabolic, and procedural factors—including diabetes, complex bifurcation anatomy, multilayer stenting, potentially incomplete lesion preparation, and the absence of intracoronary imaging during earlier procedures—coexisted and were highly interrelated. Their individual contributions to recurrent ISR therefore cannot be disentangled or causally quantified. Although subsequent IVUS objectively demonstrated severe stent underexpansion, this finding cannot establish its relative causal contribution compared with the other concomitant factors. Finally, the case does not allow direct comparison between alternative strategies such as redo CABG, exclusive DCB-based treatment or coronary brachy/laser therapy.

## 5. Conclusions

This case illustrates the challenges of recurrent revascularization failure in complex coronary disease and highlights the importance of a mechanism-based approach to recurrent ISR. Intracoronary imaging with IVUS or OCT can play an important role in identifying the underlying mechanism of stent failure, particularly mechanical factors such as stent underexpansion, and in guiding lesion preparation and procedural optimization in complex PCI. In recurrent ISR with multilayer stent architecture, careful consideration should also be given to minimizing additional metallic burden and tailoring the revascularization strategy to the anatomical and mechanical substrate.

## Data Availability

The data presented in this study are available on request from the corresponding author due to privacy reasons.

## Supplementary Materials

The following supporting information can be downloaded at: https://www.mdpi.com/article/10.3390/jcm15197710/s1, Video S1. Coronary angiography performed in September 2024 demonstrating significant two-vessel coronary artery disease. Angiographic images reveal severe stenotic lesions involving the left anterior descending (LAD) in proximal and mid segments and left circumflex (LCx) coronary arteries, identified during the evaluation of symptomatic ischemic heart disease. Video S2. Coronary angiography performed in December 2024, illustrating a complex left main bifurcation restenotic pattern (Medina 1,1,1) extending into the LAD and LCx, identified during evaluation of an acute coronary syndrome. Video S3. Sequential angiographic assessment illustrating the evolution of recurrent revascularization failure. Part 1. Coronary angiography (April 2025) demonstrating subocclusive LMCA in-stent restenosis extending into the proximal LAD and LCx, diffuse LAD disease with TIMI 2 distal flow, and ostial LCx occlusion due to ISR. Part 2. Repeat coronary and graft angiography following CABG, demonstrating persistent LMCA bifurcation ISR, ostial LCx occlusion, diffuse LAD disease, distal RIMA–OM anastomotic stenosis, and preserved LIMA–LAD graft patency. Part 3. IVUS-guided therapeutic PCI, illustrating lesion preparation, cutting-balloon and DCB treatment of the LCx, DCB treatment of the diagonal branch, LMCA DES implantation, and final post-dilatation. The sequential angiographic recordings allow dynamic assessment of vessel filling, graft function, and procedural results throughout the clinical course. Table S1. CARE Checklist.

## Abbreviations

The following abbreviations are used in this manuscript:

ISR	In-Stent Restenosis
PCI	Percutaneous Coronary Intervention
DES	Drug-Eluting Stent
LMCA	Left Main Coronary Artery
FFR	Fractional Flow Reserve
iFR	Instantaneous Wave-Free Ratio
IVUS	Intravascular Ultrasound
OCT	Optical Coherence Tomography
NT-proBNP	N-terminal pro-B-type Natriuretic Peptide
LAD	Left Anterior Descending artery
LCx	Left Circumflex artery
NSTEMI	Non-ST-Segment Elevation Myocardial Infarction
DK-crush	Double Kissing Crush
QCA	Quantitative Coronary Angiography
POT	Proximal Optimization Technique
TIMI	Thrombolysis in Myocardial Infarction
CABG	Coronary Artery Bypass Grafting
LIMA	Left Internal Mammary Artery
RIMA	Right Internal Mammary Artery
SVG	Saphenous Vein Graft
LDL	Low-Density Lipoprotein
PCSK9	Proprotein Convertase Subtilisin/Kexin Type 9
DAPT	Dual Antiplatelet Therapy
SGLT2	Sodium–Glucose Cotransporter-2
HbA1c	Glycated Hemoglobin
ESC	European Society of Cardiology
EACTS	European Association for Cardio-Thoracic Surgery
ULTIMATE	ULTIMATE Trial
IVUS-XPL	IVUS-XPL Trial
DCB	Drug-Coated Balloon
DEB	Drug-Eluting Balloon
