# Supplementary material for: Recurrent Revascularization Failure in a Patient with Complex Coronary Substrate
Source: J Clin Med. 2026 Oct 6;15(19):7710. doi: 10.3390/jcm15197710 (PMC13665949; doi:10.3390/jcm15197710)
Supplement: Supplementary file 1 [file jcm-15-07710-s001.zip › Table S1.pdf]

Supplementary Table S1. CARE Checklist

.

CARE item	CARE requirement	Status	Location / manuscript assessment
1. Title	The diagnosis or intervention of primary focus followed by “case report”.	Yes	Title identifies recurrent revascularization failure / recurrent LM bifurcation ISR after PCI and CABG and states “case report”.
2. Keywords	2-5 key words identifying diagnoses/interventions, including “case report”.	Yes	Keywords include ISR, LMCA, ischemia-guided PCI, intracoronary imaging, bifurcation disease, diabetes and revascularization failure. Add “case report” to fully satisfy CARE.
3a. Abstract - Introduction	What is unique about this case and what does it add?	Yes	Abstract background identifies recurrent ISR in complex anatomy and the importance of intracoronary imaging.
3b. Abstract - Main symptoms/findings	Main symptoms and/or important clinical findings.	Yes	Recurrent ischemic presentations, LM bifurcation ISR, graft degeneration and IVUS-detected mechanical abnormalities are summarized.
3c. Abstract - Main diagnoses/interventions	Main diagnoses, therapeutic interventions and outcomes.	Yes	Repeated PCI, CABG and final IVUS-guided hybrid DCB/DES PCI with 6- and 12-month outcome are summarized.
3d. Abstract - Conclusion	Main take-away lesson.	Yes	Mechanism-based revascularization and intracoronary imaging are emphasized.
4. Introduction	Brief background summary with relevant literature.	Yes	Introduction discusses ISR, complex LM bifurcation disease, physiology and intracoronary imaging.
5a. Patient information	De-identified demographic and other patient-specific information.	Yes	44-year-old man; no identifying personal information is reported.
5b. Main concerns and symptoms	Patient's main concerns and symptoms.	Yes	Acute constrictive retrosternal chest pain at rest lasting >30 minutes; recurrent angina during the course.
5c. Medical, family and psychosocial history	Relevant medical, family and psychosocial history, including genetic information when relevant.	Yes	Hypertension, insulin-treated type 2 diabetes and dyslipidemia are reported.
5d. Relevant past interventions	Relevant previous interventions and outcomes.	Yes	Sequential PCI, DK-crush PCI, DCB treatment, CABG and repeat PCI are described.
6. Clinical findings	Relevant physical examination and other significant clinical findings.	Yes	Hemodynamic stability, cardiovascular examination, absence of heart-failure signs and ECG/laboratory findings are described.

7. Timeline	Important information organized as a timeline.	Yes	Figure 1 plus Supplementary Figure S1. Intervals are specified: Sep-Dec 2024 (3 months), Dec-Jan (1 month), Jan-Apr (3 months), CABG Apr 2025, graft failure Apr 2025, final IVUS-guided PCI Jul 2025, then 6- and 12-month follow-up.
8a. Diagnostic assessment - methods	Diagnostic methods such as examination, laboratory testing, imaging and physiologic assessment.	Yes	ECG, biomarkers, echocardiography, angiography, FFR, QCA and IVUS are reported.
8b. Diagnostic challenges	Diagnostic challenges.	Yes	Absence of IVUS/OCT during earlier interventions and limitations of angiographic assessment are discussed.
8c. Diagnosis and differential diagnosis	Diagnosis, including other diagnoses considered when appropriate.	Yes	NSTEMI associated with severe recurrent multilayer LMCA bifurcation ISR is stated. Differential diagnosis is not a major feature of this angiographically documented presentation.
8d. Prognostic characteristics	Prognostic characteristics when applicable.	Yes	Diabetes, complex bifurcation anatomy, multilayer stenting, residual SYNTAX score and diffuse disease are described.
9a. Therapeutic intervention - type	Types of therapeutic intervention.	Yes	PCI, DES, DCB, CABG and IVUS-guided repeat PCI are described.
9b. Therapeutic intervention - administration	Administration/intensity of interventions.	Yes	Stent strategy, cutting/non-compliant balloons, kissing inflation, POT, graft configuration and secondary-prevention medication are reported.
9c. Changes in intervention	Changes in treatment and rationale.	Yes	Escalation from repeat PCI to CABG and later to IVUS-guided native-vessel PCI is explained; DCB used to limit additional metallic layers.
10a. Follow-up outcomes	Clinician- and patient-assessed outcomes.	Yes	Asymptomatic status and absence of significant angiographic restenosis at 6 and 12 months are reported.
10b. Follow-up tests	Important follow-up diagnostic and other test results.	Yes	Follow-up angiography and risk-factor control are reported.
10c. Adherence and tolerability	Intervention adherence/tolerability and how assessed.	Yes	DAPT adherence was patient-reported/clinically documented but not objectively verified.
10d. Adverse/unanticipated events	Adverse and unanticipated events.	Yes	Recurrent ISR, recurrent ACS/angina and early graft dysfunction are central to the case.
11a. Discussion - strengths/limitations	Strengths and limitations of the case.	Yes	Dedicated Limitations section; inferential mechanisms, single-patient design and limited follow-up are acknowledged.
11b. Discussion - literature	Relevant medical literature.	Yes	Discussion places ISR, intracoronary imaging, CABG/graft

			failure and DCB/DES strategy in the context of published evidence.
11c. Discussion - rationale	Rationale for conclusions, including possible causes.	Yes	Mechanistic interpretation is discussed with appropriately cautious causal language and recognition of inseparable confounding.
11d. Discussion - take-away lessons	Primary lessons from the case.	Yes	Need for intracoronary imaging, careful bifurcation optimization and mechanism-based treatment is emphasized.
12. Patient perspective	Patient should share perspective or experience whenever possible.	Not reported	No patient-perspective statement is available.
13. Informed consent	Did the patient give informed consent?	Yes	Written informed consent for publication was obtained.
